# Fertility preservation in women with endometriosis

**DOI:** 10.1093/hropen/hoaf012

**Published:** 2025-02-28

**Authors:** Antonio La Marca, Michela Semprini, Elisa Mastellari, Valeria Donno, Martina Capuzzo, Carlo Alboni, Simone Giulini

**Affiliations:** Department of Medical and Surgical Sciences for Children and Adults, University of Modena and Reggio Emilia, Modena, Italy; UO Tutela Salute Famiglia, Donna ed Età evolutiva, AUSL Romagna, Rimini, Italy; Dexeus Fertility, Department of Obstetric Gynecology and Reproductive Medicine, Hospital Universitari Dexeus, Barcelona, Spain; Tethys—Assisted Reproductive Technologies Center, Verona, Italy; Department of Medical and Surgical Sciences for Children and Adults, University of Modena and Reggio Emilia, Modena, Italy; Department of Medical and Surgical Sciences for Children and Adults, University of Modena and Reggio Emilia, Modena, Italy

**Keywords:** fertility preservation, oocyte vitrification, endometriosis, endometrioma, ovarian reserve, IVF

## Abstract

**BACKGROUND:**

Endometriosis is a chronic disease that can compromise fertility in up to 30–50% of affected patients, and it is estimated that patients affected by endometriosis represent about 10% of patients undergoing ART treatments. The hypothesized underlying mechanisms explaining infertility are various, but great attention has been given to the relationship between ovarian endometriomas and reduced ovarian reserve.

**OBJECTIVE AND RATIONALE:**

Infertility in patients with endometriosis does not have univocal management, since surgical therapy can increase the chances of natural conception, but at the same time increases the risk of damage to the ovarian reserve. In some cases, IVF procedures should be considered instead of surgery, within a personalized strategy. It has therefore been proposed that patients with endometriosis are eligible for fertility preservation.

**SEARCH METHODS:**

This article is based on a critical review of literature on peer-reviewed article indexing databases including PubMed, Scopus and Medline, using as keywords: ‘fertility preservation’, ‘oocyte vitrification’, ‘endometriosis’, ‘endometrioma’, ‘ovarian reserve’ and ‘*in vitro* fertilization’.

**OUTCOMES:**

Data regarding the feasibility of oocyte cryopreservation in patients with endometriosis have increased over recent years, indicating that these patients seem to have the same number of oocytes retrieved and IVF outcomes similar to those who perform fertility preservation for other indications. However, probably due to a reduced ovarian reserve, several cycles of ovarian stimulation may be needed to gather a suitable number of retrieved oocytes per patient. Age, ovarian reserve, and previous ovarian surgery are the main factors affecting the success of fertility preservation. Bilateral endometriomas, a history of unilateral endometrioma surgery with a contralateral recurrence, and preoperative reduced ovarian reserve are the most common indications for fertility preservation. The choice between primary surgery and ART is often complex, requiring a therapeutic strategy tailored to the patient’s clinical characteristics and needs, such as age, type and severity of endometriosis lesions, presence of symptoms, surgical history, and desire for pregnancy.

**LIMITATIONS, REASONS FOR CAUTION:**

The development of endometriosis-related infertility and the severity of ovarian damage due to endometriosis lesions *per se* or their surgical treatment are difficult to predict, and data are lacking concerning which subgroups of patients with endometriosis might benefit most from fertility preservation.

**WIDER IMPLICATIONS:**

Women with endometriosis, and in particular women with bilateral ovarian endometriomas or recurrent surgery on the ovaries, should be advised about risk of ovarian reserve damage. Oocyte cryopreservation is an established technique that has been demonstrated as feasible and successful for these patients; however, the specific indications have not yet been established.

**STUDY FUNDING/COMPETING INTEREST(S):**

There are no funding sources for the study and no conflicts of interest to declare.

WHAT DOES THIS MEAN FOR PATIENTS? Endometriosis is a condition that can harm fertility by reducing the number of available oocytes and affecting the ovaries. This review aimed to explore how fertility preservation, i.e. freezing oocytes, can help women with endometriosis maintain the possibility of having children in the future. The study reviewed recent medical research and found that techniques such as oocyte freezing are both safe and effective. However, women with severe cases or previous surgeries may need multiple treatment cycles to collect enough oocytes for preservation. Each decision, whether surgery, fertility preservation, or IVF, should be tailored to the individual’s age, health, and reproductive goals. Patients are encouraged to discuss options with a specialist to create a plan suited to their needs.

## Introduction

In recent years, the development of technologies which allow the cryopreservation of oocytes, embryos, and ovarian cortex has provided women with jeopardized ovarian reserve and reproductive capacity the possibility to preserve their fertility (Donnez and Dolmans, 2017; Rienzi *et al.*, 2017). These fertility preservation strategies were originally intended for women who need to receive gonadotoxic therapies for cancer, which might cause iatrogenic infertility (Martinez, 2017; Cobo *et al.*, 2018). However, over the years, the indications have been enlarged: today, they include age-related decline of fertility, often due to the trend of delaying childbearing in developed countries (so-called ‘social freezing’) (Stoop *et al.*, 2014; Cobo *et al.*, 2016, 2018), and various medical conditions which, even if not malignant, cause the patient to be at risk of significant loss in ovarian follicle reserve. The last group includes genetic conditions (La Marca and Mastellari, 2021) and various medical conditions, including endometriosis (Mathieu d’Argent *et al.*, 2020; Cobo *et al.*, 2021), as well as benign hematological, autoimmune, and genetic disorders (Santulli *et al.*, 2023).

The potential adoption of the proposal for fertility preservation in cases of endometriosis, as indicated by a European survey and existing literature, remains uncertain. Currently, there are no globally recognized guidelines, such as those from ESHRE or ASRM (American Society for Reproductive Medicine), which play a crucial role in disseminating and promoting the uptake of advances in reproductive medicine, specifically fertility preservation for endometriosis patients. Nevertheless, it is worth noting that as early as 2015, endometriosis was acknowledged as a potential indication for fertility preservation. A joint expert working group consisting of 20 individuals from ESHRE and ASRM emphasized the importance of discussing and providing fertility preservation options to patients with benign conditions leading to premature ovarian insufficiency, including autoimmune disorders (such as systemic lupus erythematosus, inflammatory bowel diseases, and rheumatoid arthritis), as well as gynecological conditions like endometriosis (Martinez, 2017).

### Endometriosis as a threat to spontaneous fertility

Endometriosis is a chronic inflammatory disease characterized by the presence of endometrial glands outside the uterus. It affects about 5–10% of reproductive-aged women and often causes pelvic pain, impaired fertility, or both (Giudice, 2010). Infertility is a major concern in women with endometriosis: up to 30–50% of these patients are infertile (Practice Committee of the American Society for Reproductive Medicine, 2012; Somigliana *et al.*, 2017). The prevalence of endometriosis in infertile patients, moreover, is up to 25–50%, 10 times higher than in the general population (Ozkan *et al.*, 2008; Koch *et al.*, 2012; Dunselman *et al.*, 2014); in addition, endometriosis represents 10% of the indications for performing IVF procedures (Somigliana *et al.*, 2017).

Several mechanisms have been hypothesized to explain the relationship between endometriosis and infertility. These include damage to the ovarian parenchyma caused by the presence of endometriomas, distortion of the pelvic anatomy following the formation of adhesions, chronic inflammation in the pelvis and in the peritoneal fluid associated with the presence of superficial and deep peritoneal lesions, associated adenomyosis if present, altered hormonal and cell-mediated functions in the endometrium, dyspareunia (and therefore the difficulty in having intercourse), and also possible iatrogenic damage during surgery on the ovarian parenchyma (Bulletti *et al.*, 2010; Somigliana *et al.*, 2017; Llarena *et al.*, 2019). These factors can lead to reduced tubal function, impaired folliculogenesis and/or oocyte quality, and/or alteration of the uterine microenvironment obstructing sperm movement and embryo implantation (Somigliana *et al.*, 2017; Llarena *et al.*, 2019).

Endometriosis is present as an ovarian endometrioma in 17–44% of patients (Busacca and Vignali, 2003), and particular attention in the scientific literature has been paid to the relationship between the presence of endometriomas and the alteration of the ovarian endocrine function. Endometriomas contain fluid with massive amounts of free iron, reactive oxygen species, proteolytic enzymes and inflammatory molecules, eventually leading to substitution of the normal ovarian cortical tissue with fibrous tissue followed by a reduction of the pool of primordial follicles (Matsuzaki and Schubert, 2010; Kitajima *et al.*, 2014; Sanchez *et al.*, 2014; Llarena *et al.*, 2019). A recent systematic review concluded that the presence of endometriosis significantly reduces anti-Müllerian hormone (AMH) levels compared to controls. Additionally, a subgroup analysis examined the antral follicle count (AFC) of ovaries with endometriomas and found that, before surgery, the AFC in the affected ovary is significantly lower than that in the contralateral ovary. This supports the hypothesis that most of the damage to the ovarian reserve occurs prior to surgery (Tian *et al.*, 2021). Large endometriomas can also expose the ovary to mechanical stress (Llarena *et al.*, 2019). Women with endometriomas have lower AMH levels (Sanchez *et al.*, 2014; Nieweglowska *et al.*, 2015), as noted above, as well as a more rapid decline in ovarian reserve than compared with age-matched non-endometriosis controls (Kasapoglu *et al.*, 2018). Moreover, an increased risk of premature ovarian failure (Kitajima *et al.*, 2014) and less ovulation in the affected ovary compared with the normal ovary (Horikawa *et al.*, 2008) are often reported with endometriosis. Reduced oocyte quality in women with endometriomas has also been suggested, although these data are still controversial. Data from IVF procedures show that women with endometriosis have oocytes with lower *in-vitro* maturation rates and lower fertilization rates (Barnhart *et al.*, 2002; Harb *et al.*, 2013; Sanchez *et al.*, 2017).

Several studies have explored the quality of embryos originating from oocytes of women with endometriosis to assess whether endometriosis impacts embryo quality. In a study involving 235 human embryos, researchers found that nuclear and cytoplasmic abnormalities, cytoplasmic fragmentation, and uneven cleavage were more commonly observed in embryos derived from oocytes of women with endometriosis compared to those from patients with different types of infertility (Brizek *et al.*, 1995). Nevertheless, the embryo aneuploidy rate seems to be similar in patients with endometriosis and unaffected age-matched controls (Juneau *et al.*, 2017), and affected patients seem to have similar pregnancy, live birth, and miscarriage rates in comparison with those who have undergone IVF for other reasons (González-Comadran *et al.*, 2017). Although the relationship between endometriosis and infertility is supported by substantial evidence, only 30–50% of women with endometriosis are actually infertile, and many affected patients can achieve natural conception (Practice Committee of the American Society for Reproductive Medicine, 2012; Somigliana *et al.*, 2017).

### The role of surgery in improving fertility

Surgical treatment of endometriosis-associated infertility is a controversial topic. In particular, it is debated whether the first approach in these patients should be surgery or IVF (Calagna *et al.*, 2020). The decision-making process is complex, since the possibility of improving natural fertility through surgical intervention can be conditioned by several factors, i.e. the presence and degree of pain symptoms, patient age and preferences, history of prior surgeries, presence of other infertility factors, ovarian reserve, and the estimated endometriosis fertility index (EFI). This staging system forecasts non-IVF pregnancy rates subsequent to surgical endometriosis staging and treatment. Before surgery, the diagnosis of deep infiltrating endometriosis (DIE) can also serve as a valuable predictor of surgical complexity. Additionally, in the context of infertility, the presence of DIE plays a crucial role in determining whether to proceed with a surgical intervention or opt for ARTs, particularly when combined with predictive tools like the EFI (Condous *et al.*, 2024). The first surgical indication for patients with endometriosis is the presence of symptoms which cannot be optimally managed by medical therapy (Dunselman *et al.*, 2014). However, also in asymptomatic patients, it is reported that surgery can increase the chances of infertile couples of achieving a natural pregnancy. In patients with ASRM stage I and II endometriosis, some randomized controlled trials show that excision or ablation of endometriotic lesions and lysis of adhesions during laparoscopic surgery increases natural pregnancy rates in comparison with only diagnostic laparoscopy (Marcoux *et al.*, 1997; Parazzini, 1999; Moini *et al.*, 2012; Duffy *et al.*, 2014). For these reasons, several guidelines (Koch *et al.*, 2012; Practice Committee of the American Society for Reproductive Medicine, 2012; Dunselman *et al.*, 2014) advocate for laparoscopic intervention that includes ablation or excision of all visible endometriosis lesions as a preferred approach over diagnostic laparoscopy alone. Each guideline highlights different facets of why this approach is beneficial. Koch *et al.* (2012) emphasize the enhancement of natural pregnancy rates, while the Practice Committee of the American Society for Reproductive Medicine (2012) focuses on the importance of fertility treatment. Dunselman *et al.* (2014), on the other hand, offer a comprehensive perspective that includes both symptom management and fertility preparation. In summary, these guidelines collectively support the use of therapeutic laparoscopy for managing endometriosis, demonstrating its crucial role in enhancing patient outcomes overall.

The efficacy of surgical treatment in improving fertility is less clear in ASRM stage III and IV endometriosis (Koch *et al.*, 2012; Dunselman *et al.*, 2014). A prospective cohort study by Adamson and Pasta (1994) found a 3-year estimated cumulative pregnancy rate of 62% after laparoscopic surgery in these patients. Other prospective cohort studies suggest a significant improvement in pregnancy rates after IVF in patients who were surgically treated for DIE before the IVF procedures compared with non-operated patients (Ferrero *et al.*, 2009; Barri *et al.*, 2010; Daraï *et al.*, 2010). Liang *et al.* (2024) showed that surgery, whether complete or incomplete, resulted in improved pregnancy rates per patient. This finding highlights the significance of surgical interventions as a treatment for DIE; even if the surgery does not entirely eliminate the disease, it can still offer considerable benefits in terms of fertility outcomes. However, the decision to undergo surgery should always be weighed against its potential risks, and these should be carefully discussed with patients.

In contrast, some other studies have shown no improvement in fertility outcomes with surgery (Vercellini *et al.*, 2006). Thus, in women with DIE, laparoscopic intervention could be proposed to restore normal pelvic anatomy, but the indication should be carefully discussed with the patient (Dunselman *et al.*, 2014; Kuznetsov *et al.*, 2017). Some experts suggest that IVF, rather than surgery, should be the first-line treatment for women with DIE who primarily desire childbearing (Abrão *et al.*, 2015; Falcone and Flyckt, 2018).

Some data support the surgical removal of ovarian endometriomas in order to improve natural conception rates (Hart *et al.*, 2008; Llarena *et al.*, 2019). In a survey, half of the respondents (28 centers, 48.3%) allowed pregnancy immediately after surgery, 24 centers (41.4%) would recommend waiting for at least 3 months, and six centers recommended waiting up to 6 months before pregnancy (Sänger *et al.*, 2023). Guidelines recommend surgical excision by stripping, which decreases the risk of recurrence and increases the probability of natural pregnancy, compared to the drainage and electrocoagulation technique (Hart *et al.*, 2008; Dunselman *et al.*, 2014; Kuznetsov *et al.*, 2017).

### Impact of surgical approaches on ovarian reserve and fertility preservation in endometriosis management

When an endometrioma is removed to increase fertility, the potential advantage must be balanced with the risk (described by several authors) of causing damage to the ovarian reserve (Dunselman *et al.*, 2014). In fact, many studies and meta-analyses have shown that AMH levels decrease after endometrioma excision (Ragni *et al.*, 2005; Li *et al.*, 2009; Chang *et al.*, 2010; Raffi *et al.*, 2012; Somigliana *et al.*, 2012; Seyhan *et al.*, 2015; Goodman *et al.*, 2016). Moreover, ovarian cystectomy has been associated with a 2.4–13% risk of premature ovarian failure in the post-operative phase (Busacca *et al.*, 2006; Benaglia *et al.*, 2010).

Advanced age, elevated BMI, shorter menstrual cycles, bilateral ovarian cysts, advanced surgical staging, or a fully enclosed Douglas pouch were all associated with significantly lower AMH levels prior to treatment compared to individuals without these conditions. Moreover, AMH levels showed an additional decline within 1 year following laparoscopic cystectomy (Zhang *et al.*, 2024). Various mechanisms contributing to surgery-related damage to the ovarian parenchyma have been proposed, including the excessive removal of healthy ovarian tissue (Hachisuga and Kawarabayashi, 2002; Muzii *et al.*, 2002), vascular damage resulting from electrocoagulation, and autoimmune responses triggered by intense local inflammation (Garcia-Velasco and Somigliana, 2009; Li *et al.*, 2009). Conservative surgical management of ovarian endometriomas typically involves several options, including cystectomy by stripping, various ablative techniques (such as those using laser, plasma energy, or bipolar diathermy), and sclerotherapy with ethanol, as well as combined approaches.

In cystectomy by stripping, the process starts with draining the endometrioma, followed by the careful separation of the cyst wall from the ovarian cortex using gentle traction and counter-traction. Hemostasis is then achieved at the cyst bed, often utilizing bipolar diathermy, sutures or hemostatic agents. Both the suture method and hemostatic agents may have potential benefits in the preservation of ovarian reserve over the bipolar coagulation method when cystectomy for ovarian endometrioma is performed (Paik and Jee, 2024). Ablative techniques involve fenestrating, draining, and washing out the cyst, followed by the destruction of the ovarian endometrioma cyst wall (Muzii *et al.*, 2007). Compared to bipolar diathermy, laser and plasma energy provide a more superficial, tissue-conserving effect, thereby minimizing unintended damage to the underlying ovarian tissue (Pados *et al.*, 2010; Roman *et al.*, 2011). Both ablation and cystectomy have significantly detrimental effects on the ovarian reserve as evaluated by AMH, but the ablation causes relatively less damage to ovarian reserve when appraised by AFC (Zhang *et al.*, 2022).

For larger endometriomas, a ‘three-stage procedure’ may be utilized. This approach begins with the initial drainage of the cyst during laparoscopy, followed by 12 weeks of treatment with GnRH agonists to reduce the cyst size, and concludes with laser vaporization of the cyst wall during a second laparoscopic procedure (Pados *et al.*, 2010).

In 2010, Donnez *et al.* described a combined approach that involves removing 80–90% of the endometrioma via cystectomy, with the remaining 10–20% near the hilus being treated with laser ablation. This method aims to minimize the accidental loss of ovarian follicles (Donnez *et al*., 2010).

Sclerotherapy for endometriomas during laparoscopy involves puncturing the cyst, aspirating its contents, and then exposing the cyst wall to a 96% alcohol (ethanol) solution for 10–15 min (Crestani *et al.*, 2022). Historically, cystectomy has been considered the gold-standard surgical approach due to its lower risk of endometrioma recurrence, reduction in endometriosis-related pain (Becker *et al.*, 2022), and higher chances of natural conception (Hart *et al.*, 2008). However, since ovarian cystectomy has also been associated with a 2.4–13% risk of premature ovarian failure in the post-operative phase (Busacca *et al.*, 2006; Benaglia *et al.*, 2010), alternative solutions have been developed to address this concern. Additionally, the expertise of the surgeon is crucial in these procedures, as it significantly impacts the preservation of ovarian function and the overall success of the surgery.

The reduction in the ovarian reserve after surgery is unpredictable (Vercellini *et al.*, 2013); however, the post-operative serum AMH level decreases more in bilateral endometrioma than in unilateral endometrioma patients (Busacca *et al.*, 2006; Chang *et al.*, 2010; Falcone and Flyckt, 2018), not surprisingly.

Some possible measures to reduce ovarian damage during surgery have been described, including, for example, the careful delineation of the plane between the endometrioma and the ovarian cortex, the sparing use of electrocoagulation for hemostatic purposes, and the use of suturing or hemostatic agents in order to minimize electrosurgery (Llarena and Flyckt, 2017; Llarena *et al.*, 2019). In this context, the experience of the surgeon seems to play a critical role in preserving fertility after surgery (Yu *et al.*, 2010).

Endometriosis is nevertheless a chronic disorder that tends to recur, with a risk for the patient of needing to undergo recurrent surgery. Repeated surgery for endometriosis does not appear to further improve fertility outcomes and often results in a greater decline in AMH than the initial surgery (Vercellini *et al.*, 2013; Ferrero *et al.*, 2015; Muzii *et al.*, 2015).

### The role of ART for patients with endometriosis

While the availability of fertility counseling for a patient might be influenced by the type (surgeon or fertility specialist) and the expertise of the initial physician she is referred to, the role of ART in endometriosis patients’ management is nonetheless still debated. It is estimated that patients affected by endometriosis represent about 10% of patients undergoing ART treatments (Somigliana *et al.*, 2017).

ART should be the first intention in treatment of infertile patients with ovarian endometriomas, to avoid surgical damage to the ovarian reserve before treatment (Lessey *et al.*, 2018), and in patients with DIE who primarily desire childbearing (Abrão *et al.*, 2015; Falcone and Flyckt, 2018). Current guidelines recommend considering the removal of asymptomatic endometriomas before IVF only in case of large endometriomas (>4 cm) to improve access to follicles during oocyte retrieval (Practice Committee of the American Society for Reproductive Medicine, 2012; Dunselman *et al.*, 2014).

The most widely agreed indications for ART in the scientific literature concern asymptomatic infertile patients over 35 years of age or with decreased ovarian reserve, other associated infertility factors, bilateral endometriomas, endometrioma recurrence, previous history of surgery for endometriosis, or failure of natural conception after surgery (Practice Committee of the American Society for Reproductive Medicine, 2012; Dunselman *et al.*, 2014; Somigliana *et al.*, 2017; Lessey *et al.*, 2018).

Ovarian stimulation with GnRH agonists or antagonists can be performed without contraindications in endometriosis patients, as they do not increase the risk of disease progression (Benaglia *et al.*, 2010, 2011; Dunselman *et al.*, 2014). The decline in fertility among women with endometriosis is primarily associated with quantitative rather than qualitative impairments. Initially, ovarian stimulation involved prolonged pituitary suppression with GnRH agonists, but this approach was swiftly replaced by GnRH antagonists due to their advantages, including shorter treatment duration and reduced gonadotropin doses. Current research supports the interchangeability of both options, as they result in similar numbers of retrieved oocytes and pregnancy rates. Additionally, it is prudent to contemplate PPOS (progestin-primed ovarian stimulation) protocols for cycles where a fresh embryo transfer is not planned (Goyri *et al*., 2024).

There are conflicting data regarding the fact that the presence of endometriotic lesions can negatively affect the results of ART; some meta-analyses report no influence (Barbosa *et al.*, 2014), and others report a negative influence dependent on the stage of the disease, with a success rate significantly lower for patients with severe disease (Barnhart *et al.*, 2002; Hamdan *et al.*, 2015). In particular, a reduction of up to 30% for clinical pregnancy rate and up to 40% for live birth rate (LBR) are reported in case of stage III or IV endometriosis (Hamdan *et al.*, 2015). A further meta-analysis (Rossi and Prefumo, 2016) found a lower pregnancy rate, but a similar LBR, compared to the controls.

### New horizons in endometriosis diagnosis and management: the role of biomarkers

Despite the exploration of various non-invasive diagnostic approaches, including the analysis of blood, cervicovaginal fluid, and urine, a definitive biomarker for endometriosis has yet to be identified. Extensive research into blood and urine tests, alongside investigations of altered cytokines, angiogenic factors, and growth factors, has not yielded a conclusive diagnostic tool for this condition (Nisenblat *et al.*, 2016; Anastasiu *et al.*, 2020; Dolińska *et al.*, 2023; Karkia *et al.*, 2022).

In a recent Cochrane review (Nisenblat *et al.*, 2016), none of the 60 investigated peripheral blood biomarkers showed sufficient accuracy to serve as a replacement test (sensitivity ≥94%, specificity ≥79%), as a triage test to rule out endometriosis in cases of a negative result (sensitivity ≥95%, specificity ≥50%) or to determine endometriosis for a positive result (sensitivity ≥50%, specificity ≥95%) in a minimally invasive manner.

The most consistently studied glycoprotein in endometriosis has been CA-125, which is increased in many physiological and pathological conditions. A meta-analysis conducted 20 years ago revealed elevated CA-125 levels in patients with endometriosis, suggesting a potential link to the more advanced stages of the disease. Subsequent studies have indicated that while CA-125 is a biomarker for endometriosis, its levels fluctuate throughout the menstrual cycle and can differ based on the clinical type and stage of the condition. Among the biomarkers studied, only four (anti-endometrial autoantibodies (anti‐endometrial Abs), interleukin‐6 (IL‐6), CA‐19.9 and CA‐125) had sufficient research support to allow a meaningful evaluation of their diagnostic accuracy. However, none of these markers proved reliable enough to become a diagnostic test (Nisenblat *et al.*, 2016).

In endometriosis, cytokines are supposed to play a key role in the decreased immunological surveillance, contributing to implantation of endometriotic foci. The most studied ones were IL-6 and TNF-α, and in 2002, Bedaiwy *et al.* (2002) suggested that they might be used as endometriosis biomarkers. In 2006, Martinez *et al.* (2007) found that women with minimal to mild endometriosis had higher serum levels of IL-6 compared to other groups, and using the cut-off level of 25.75 pg/ml, the authors described a significant accuracy of IL-6 in detecting endometriosis (sensitivity 75.0%, specificity 83.3%). However, no cytokine has been demonstrated to be sufficiently reliable as a biomarker for endometriosis. The issue lies in their lack of specificity, as these molecules can be implicated in various biological processes, both physiological (such as the menstrual cycle) and pathological.

Marí-Alexandre *et al.* (2018) were the first to characterize the miRNA profile of peritoneal fluid of women with endometriosis and to examine how miRNA levels correlate with fertility status. Their study identified a total of 126 miRNAs with altered expression in the peritoneal fluid of endometriosis patients: 78 were downregulated and 48 were upregulated. Notably, higher levels of miR-106b-3p, miR-451a, and miR-486-5p were observed in the peritoneal fluid of infertile women with endometriosis, indicating that these miRNAs might serve as potential biomarkers for predicting fertility outcomes in these individuals (Marí-Alexandre *et al.*, 2018). More recently, another study introduced a saliva-based miRNA signature profile as a novel diagnostic tool for screening endometriosis-related infertility (Dabi *et al.*, 2023).

Numerous studies have indicated that nanoparticles (i.e. materials measuring less than 100 nm in size) show great potential for advancing diagnostic and imaging technologies. These improvements could facilitate more precise non-invasive detection, a deeper understanding of target signaling pathways, and the discovery of therapeutic options for a wide range of diseases (Patra *et al.*, 2018; Moses *et al.*, 2021; Erdil, 2024; Huseynov *et al.*, 2024).

The identification of specific biomarkers, like aberrantly expressed miRNAs and inflammation-associated interleukins, could enable earlier and more accurate diagnosis. Early detection is crucial, as it allows for intervention before the disease progresses to more severe stages, significantly improving the patients’ quality of life and reducing their risk of long-term complications. Moreover, a deeper understanding of inflammatory pathways and biomarkers can lead to more personalized therapies. Treatments can be tailored based on an individual’s biomarker profile, enhancing the effectiveness of therapy while minimizing side effects.

Additionally, biomarkers may be useful not only for diagnosis and treatment but also for monitoring disease progression and assessing treatment response. Tracking changes in biomarker levels can provide valuable insights into how well a patient is responding to treatment and whether adjustments to the therapeutic plan are needed. In summary, integrating new findings on biomarkers into the diagnostic and therapeutic approach for endometriosis has the potential to revolutionize disease management. This could facilitate earlier and more accurate diagnosis, enable more targeted and personalized treatment, and ultimately improve overall patient care and quality of life.

### Emotional and psychological impact of endometriosis on fertility: insights and implications

It is well established that both potential and diagnosed infertility can impose significant emotional and financial stress. Research indicates that women experiencing infertility benefit greatly from supportive care and timely treatment. However, there is limited understanding of how endometriosis affects the lives of women, perceptions of symptom onset, disease progression, and its impact on fertility. Women with endometriosis face various choices related to family planning and fertility, including elective oocyte vitrification, surgical removal of endometriomas, and ARTs. A survey by Navarria-Forney *et al.* (2020) explored fertility issues among women aged 18–40 with endometriosis, revealing that most women (96%) were concerned about how endometriosis would affect their fertility. About half (52%) felt adequately informed by their doctors, while 31% sought more information. Despite this, only 27% considered themselves well-informed about fertility preservation options. Specialist-provided information on endometriosis and reproduction was rated as most useful, followed by patient support groups, whereas information from general gynecologists was less valued. Many women would consider ART (74%) or adoption (70%) if faced with infertility, with 72% were open to oocyte vitrification for preservation but only 37% were open to oocyte donation. Women’s stories emphasize that fertility issues are inseparable from the lived experience and the complexities of endometriosis-related impacts on both the individual and the couple. Furthermore, there is a challenging dynamic between women with endometriosis and their healthcare providers. Women’s accounts describe the trivialization of pain, disinterest, and a lack of medical knowledge, as well as inadequate information provision and medical wandering without a diagnosis or sufficient treatment (Ng *et al.*, 2020; Girard *et al.*, 2023). A patient-centered approach to investigation enables the exploration of a patient’s perceptions and beliefs about their illness, providing insight into how the various effects of endometriosis influence fertility and family planning. Exploring perceptions of the patient’s illness involves training in communication skills and education to understand and engage with their personal experiences and perspectives (Petrie *et al.*, 2007).

### Should fertility preservation have a role?

In recent years, interest in reproductive options for patients with risk of future reproductive capacity impairment has grown considerably, and the opportunity to preserve fertility has been expanded also to non-cancer patients (Garcia-Velasco *et al.*, 2013; Calagna *et al.*, 2020). Today, fertility preservation options for patients include the cryopreservation of oocytes, embryos, or ovarian tissues, which is no longer considered an experimental procedure (Practice Committee of the American Society for Reproductive Medicine, 2019).

Fertility preservation has been proposed in pre-surgical counseling for women with endometriosis at reproductive ages if they have not yet completed family planning and have negative prognostic factors in terms of fertility (Carrillo *et al.*, 2016; Practice Committee of the American Society for Reproductive Medicine, 2019). ESHRE guidelines on fertility preservation considered benign disorders to be an indication for fertility preservation but did not address whether endometriosis was a reason for fertility preservation in particular (Becker *et al.*, 2022).

International literature on the topic is poor, and no definitive and shared guidelines exist on indications and recommendations for fertility preservation in patients with endometriosis, which is still debated among experts. The aim of this review is to summarize the current evidence on fertility preservation for patients affected by endometriosis.

## Search methods

This article is based on a critical review of literature on peer-reviewed article indexing databases, including PubMed, Scopus, and Medline, using the keywords: ‘fertility preservation’, ‘oocyte vitrification’, ‘endometriosis’, ‘endometrioma’, ‘ovarian reserve’, and ‘*in vitro* fertilization’.

## Outcomes

In 2020, Calagna *et al.* published the first systematic review describing the applications of fertility preservation techniques for patients suffering from endometriosis (Calagna *et al.*, 2020). In most of the reported studies, fertility preservation happened through oocyte cryopreservation. The first case report in the review reported oocyte cryopreservation for a 25-year-old nulliparous woman who had undergone previous unilateral salpingo-oophorectomy due to ovarian endometriosis and, at the time of the study, showed reduced ovarian reserve markers (AFC 3 in the remaining ovary). After three cycles of ovarian stimulation, 21 mature oocytes (MII) were cryopreserved (Elizur *et al.*, 2009). Subsequently, Garcia-Velasco *et al.* (2013) performed a retrospective multicenter observational study regarding oocyte vitrification outcomes for fertility preservation in oncological versus non-oncological patients, including the results of fertility preservation in 38 endometriosis patients. After one cycle of ovarian stimulation using a GnRH antagonist protocol, all patients obtained MII oocytes (9.9–22.6 oocytes per patient). The authors reported that five patients with endometriosis returned to use their vitrified oocytes, but outcomes were not specified (Garcia-Velasco *et al.*, 2013). It is important to note two factors regarding endometriomas: their size and bilaterality. Studies involving women with unilateral endometriomas undergoing IVF cycles revealed that both the affected ovary and the healthy ovary produced a similar number of codominant follicles and oocytes (Filippi *et al.*, 2014). Conversely, women with bilateral endometriomas experienced an even lower ovarian response; however, pregnancy rates per transfer remained unaffected (Karadağ *et al.*, 2020). Nonetheless, the cumulative LBR might be lower due to the diminished ovarian response, underscoring the quantitative impact of the disease on fertility (Goyri *et al.*, 2024).

Only in 2018, Raad *et al.* (2018) published a retrospective observational study describing the first cohort of 49 endometriosis patients who underwent oocyte vitrification for fertility preservation. The mean number of retrieved and vitrified oocytes was, respectively, 9.5 (±6.1) and 7.2 (±4.9). No differences were noted in the mean duration of stimulation and total dose of gonadotropin used between patients with different endometriosis phenotypes (superficial versus deep infiltrated endometriosis versus ovarian endometrioma) or different surgical histories. The average number of oocytes retrieved and vitrified per cycle was significantly lower in patients with a history of endometrioma excision compared to those who had not undergone prior ovarian surgery (Raad *et al.*, 2018). A larger number of patients was reported in the retrospective observational study by Cobo *et al.* (2020). Inclusion criteria were patient age up to 42, ovarian endometrioma larger than 1 cm (with and without deep endometriosis), healthy residual ovarian tissue, and AMH level >0.5 ng/ml. Data were reported for 485 patients out of 1044 who returned to use their vitrified oocytes in an attempt to achieve pregnancy. The average number of oocytes vitrified per patient was 9.4, with significantly higher numbers of oocytes vitrified per cycle in women without prior ovarian surgery before fertility preservation. Additionally, none of the evaluated parameters (such as the number of retrieved and MII vitrified oocytes, embryo quality, survival rate, pregnancy rate, and cumulative LBR) showed statistically significant differences between early-stage (I–II) and advanced-stage (III–IV) endometriosis. They found oocyte survival rates of 83.2% after warming and a cumulative LBR of 46.4%, and concluded that fertility preservation was a valid treatment option in patients with endometriosis. However, the same parameters were statistically better in patients ≤35 years old at the time of oocyte vitrification compared with older patients. Only one study regarding embryo cryopreservation was reported in Calagna *et al.*’s review (Calagna *et al.*, 2020). This was a retrospective cohort study on 39 infertile patients with diminished ovarian reserve, among whom 16 women had endometriosis. Ovarian stimulation was performed before laparoscopic ovarian cystectomy using the stripping method. Although few data on outcomes were specified, the authors reported that six endometriosis patients experienced live birth after successive embryo transfer (Kuroda *et al.*, 2019).

Only three case reports were reported by Calagna *et al.* (2020) on ovarian tissue cryopreservation. In two cases, endometrioses was the main indication for fertility preservation. The first article reported two cases of nulliparous young patients previously operated for endometrioma, with recurrent severe endometriosis (including 8–9 cm endometriomas); because of the severity of the disease, these patients had unilateral oophorectomies, but during surgery, fresh 10–12 mm strips of ovarian cortex were reimplanted in a peritoneal window near the contralateral ovarian hilus (orthotopic reimplantation). In both cases, the grafted tissue showed the presence of ovarian activity (i.e. presence of follicles) at laparoscopic follow-up, and one patient became pregnant through IVF (Donnez *et al.*, 2005). The presence of ovarian activity up to 9 months after the graft was also reported within Oktay and Oktem’s series, in a case of orthotopic transplantation of ovarian cortex frozen 6 months before in a patient with endometriosis (Oktay and Oktem, 2010).

Some other studies have provided additional information on the topic. Kim *et al.* (2020) performed a retrospective evaluation on oocyte cryoconservation for fertility preservation in women with ovarian endometrioma (but without DIE) who were scheduled to undergo ovarian cystectomy. Patients were counseled to consider fertility preservation before surgery in case of AMH values lower than expected for their age or less than 3.0 ng/ml, or bilateral endometriomas or recurrent endometrioma. Only 34 of 68 patients (50%) agreed to undergo oocyte cryoconservation after counseling. The number of oocytes retrieved, but not the number of MII oocytes retrieved, was significantly lower in the patients with endometrioma. In 13 patients (38.2%), it was necessary to repeat ovarian stimulation and oocyte retrieval to obtain an adequate number of oocytes to cryopreserve, where 10 MII oocytes was the goal of the stimulation. Patients with bilateral endometriomas had lower numbers of oocytes cryopreserved compared to patients with unilateral endometrioma (4.1 ± 2.9 versus 5.7 ± 3.4); the difference was, however, not statistically different. Patients with endometrioma received higher total doses of gonadotropins compared with those who did not have ovarian endometriosis, probably due to lower AMH levels. There was no change in size of the endometriomas before and after ovarian stimulation. Outcomes in terms of pregnancy rate and LBR were not reported (Kim *et al.*, 2020).

Two non-randomized studies comparing PPOS versus GnRH antagonist protocols reported similar numbers of retrieved oocytes in both groups (Guo *et al.*, 2020; Mathieu d’Argent *et al.*, 2020), suggesting that the PPOS protocol could serve as a viable alternative for women with endometriosis, particularly when a fresh embryo transfer is not planned; the rationale behind using progestins lies in their comparable efficacy to GnRH antagonists in preventing premature LH surges. From a practical standpoint, a woman already undergoing progestin treatment to manage her symptoms could seamlessly transition to gonadotropin stimulation and continue the same progestin regimen.

Fouks *et al.* (2023), in an observational cross-sectional study, identified a significant negative correlation between the number of clinical symptoms and the quantity of vitrified oocytes. This association was only partially linked to prior surgical interventions. Among patients with endometriosis undergoing fertility preservation, AMH levels showed the strongest correlation with treatment success. Cobo *et al.* conducted a retrospective observational study in order to investigate how the number of oocytes retrieved affects the cumulative live birth rate (CLBR) in endometriosis patients who had their oocytes vitrified for fertility preservation (Cobo *et al.*, 2021). A total of 485 women with endometriosis (stage III–IV endometriosis in about 98% of cases, before or after surgery) were included. The study confirmed that, also in patients with endometriosis, the LBR is positively correlated with the number of oocytes stored and patient age: better outcomes were observed in young women (aged ≤35 years old versus >35 years old). The LBR was 95.4% in the younger group versus 79.6% in older women, using approximately 20 oocytes. This finding highlights the relevance of oocyte vitrification at a young age also in women with endometriosis. Moreover, comparing the results in this endometriosis group with those in women who underwent fertility preservation for other causes (Cobo *et al.*, 2018), no statistical differences in age-matched groups with the same number of vitrified oocytes were observed. When the same number of oocytes was vitrified, the overall CLBR in endometriosis patients showed no statistical differences compared to those who performed elective fertility preservation, irrespective of age. On the basis of these observations, the authors suggested that ovarian endometriosis and previous ovarian surgery for endometriosis have a quantitative and not qualitative negative effect on IVF outcomes. Probably due to a reduced ovarian reserve, in fact, endometriosis patients showed a lower number of oocytes retrieved per cycle compared to patients who were not affected. However, if an optimal number of oocytes was retrieved, no impairment in LBR in IVF cycles was seen. The results of the study do not demonstrate a compromised LBR in IVF cycles (Cobo *et al.*, 2021).

In contrast, a recent meta-analysis by Liang *et al.* (2024) found that the pregnancy rate per patient (OR 1.47; 95% CI, 0.59–3.63), pregnancy rate per cycle (OR 1.16; 95% CI, 0.45–2.99), and live birth per patient (OR 1.66; 95% CI, 0.56–4.91) were similar in patients with DIE, whether treated with surgery or ART as the initial approach. However, when cases of both complete and incomplete surgical excision of DIE were included, surgery was associated with a significant improvement in the pregnancy rate per patient (OR 1.63; 95% CI, 1.11–2.40).

## Discussion

### Oocyte cryopreservation: a feasible option for endometriosis patients

Among the various techniques reported for fertility preservation, oocyte cryopreservation has been the most frequently proposed in literature in case of endometriosis. Oocyte cryopreservation does not have a negative impact on ovarian reserve, has low associated morbidity, and does not require a male partner (Donnez and Dolmans, 2017). For these reasons, it seems to be the most suitable technique for young patients with regular menstrual cycles but suffering from a benign pathology that can potentially compromise the ovarian reserve in the future (Carrillo *et al.*, 2016). Studies like Cobo *et al.* (2016) report oocyte survival rates of 85.2%, with LBRs depending on age and the number of oocytes retrieved.

### Multiple ovarian stimulation cycles may be needed to achieve optimal results

Due to a reduced ovarian reserve in endometriosis patients, multiple stimulation cycles may be required to retrieve an optimal number of oocytes (at least 10) (Carrillo *et al.*, 2016; Kim *et al.*, 2020; Mathieu d’Argent *et al.*, 2020; Cobo *et al.*, 2021). For instance, Raad *et al.* (2018) and Cobo *et al.* (2020) reported an average of approximately 9 vitrified oocytes per patient, while those with severely compromised reserve vitrified only 4–6 oocytes on average.

### Age and ovarian reserve are critical predictors of success

Age and ovarian reserve are key determinants of fertility preservation outcomes. Cobo *et al.* (2021) found that CLBRs increased significantly with a higher number of cryopreserved oocytes but decreased with advanced age.

### Surgical history and stage of disease influence oocyte retrieval

Surgical history and disease stage impact the number of retrieved oocytes. Patients with prior ovarian surgery have fewer oocytes retrieved than those without surgical history (Raad *et al.*, 2018; Cobo *et al.*, 2020; Santulli *et al.*, 2021; Elizur *et al.*, 2023). On the other hand, the stage of the disease does not seem to significantly influence the number of cryopreserved oocytes (Raad *et al.*, 2018; Cobo *et al.*, 2020; Kim *et al.*, 2020; Mathieu d’Argent *et al.*, 2020; Cobo *et al.*, 2021). As previously mentioned, some prospective cohort studies seem to suggest a significant improvement in pregnancy rates in patients with DIE that is surgically treated before the IVF procedures, compared with non-operated patients (Ferrero *et al.*, 2009; Barri *et al.*, 2010; Daraï *et al.*, 2010).

### Comparable IVF outcomes with adequate oocyte retrieval

When an adequate number of oocytes is cryopreserved, IVF outcomes are comparable to those in patients who underwent fertility preservation for other reasons. Cobo *et al.* (2021) showed similar LBRs between endometriosis patients and age-matched controls with an equal number of oocytes.

### The importance of fertility preservation before surgery

Fertility preservation before surgery can protect the ovarian reserve from the detrimental effects of surgical interventions. Studies like Llarena *et al.* (2019) highlight the benefits of this approach for patients with bilateral endometriomas or a preoperative diminished ovarian reserve.

### Fertility preservation tailored to patient characteristics

Fertility preservation should be tailored based on patient-specific factors, such as age, ovarian reserve, presence of bilateral endometriomas, or history of repeated surgeries. The indications for fertility preservation in patients with endometriosis, as highlighted by the analyzed studies (see Table 1), include several key factors. The patient’s age is one of the main considerations (Carrillo *et al.*, 2016; Llarena *et al.*, 2019; Mathieu d’Argent *et al.*, 2020; Carneiro *et al.*, 2021; Fouks *et al.*, 2023; Mifsud *et al.*, 2023; Sänger *et al.*, 2023; Goyri *et al.*, 2024), particularly after the age of 35, when fertility may begin to decline more significantly (Cobo *et al.*, 2020). The presence of bilateral endometriomas is another relevant indication (Somigliana *et al.*, 2015; Carrillo *et al.*, 2016; Llarena *et al.*, 2019; Kim *et al.*, 2020; Pluchino and Roman, 2020; Carneiro *et al.*, 2021; Elizur *et al.*, 2023; Mifsud *et al.*, 2023; Goyri *et al.*, 2024). Additionally, a history or risk of multiple surgeries may suggest the need for fertility preservation due to the potential compromise of ovarian function (Llarena *et al.*, 2019; Cobo *et al.*, 2020; Mathieu d’Argent *et al.*, 2020; Santulli *et al.*, 2021; Elizur *et al.*, 2023). A reduced preoperative ovarian reserve (Carrillo *et al.*, 2016; Llarena *et al.*, 2019; Pluchino and Roman, 2020; Fouks *et al.*, 2023; Goyri *et al.*, 2024), as well as the combination of ovarian endometrioma and reduced ovarian reserve (Mathieu d’Argent *et al.*, 2020; Kim *et al.*, 2020; Carneiro *et al.*, 2021; Fouks *et al.*, 2023; Mifsud *et al.*, 2023; Goyri *et al.*, 2024), are further factors that may indicate the need for fertility preservation. Finally, in patients who have already undergone surgery for unilateral endometrioma and/or present with contralateral recurrence before a new surgery, fertility preservation may be particularly recommended (Somigliana *et al.*, 2015; Carrillo *et al.*, 2016; Llarena *et al.*, 2019; Carneiro *et al.*, 2021; Mifsud *et al.*, 2023).

**Table 1.** Indications for fertility preservation.

Indications
Age over 35 years
Bilateral endometriomas, before surgery
History or risk of multiple surgery
Preoperative reduced ovarian reserve
Ovarian endometrioma and reduced ovarian reserve
Previous unilateral endometrioma surgery
Recurrent endometrioma, before surgery
Severe endometriosis diagnosis
Ovarian endometrioma larger than 4 cm
Unlikely postoperative natural conception
Severe cases identified via surgery
Low EFI score
Endometriosis stages III and IV

Indications for fertility preservation of the papers included in this review are listed in order of prevalence. Somigliana *et al.* (2015), Carrillo *et al.* (2016), Llarena *et al.* (2019), Pluchino and Roman (2020), Mathieu d'Argent *et al.* (2020), Kim *et al.* (2020), Cobo *et al.* (2020), Santulli *et al.* (2021), Elizur *et al.* (2023), Carneiro *et al.* (2021), Fouks *et al.* (2023), Goyri *et al.* (2024), Mifsud *et al.* (2023), Sänger *et al.* (2023).

EFI: endometriosis fertility index.

### Additional considerations for fertility preservation

Embryo cryopreservation is a highly effective technique; however, it requires the involvement of a male partner, which can lead to ethical and legal concerns, particularly regarding the fate of embryos in cases of the patient’s death or separation from her partner. Among all fertility preservation methods, oocyte cryopreservation is considered the preferred option for post-pubertal patients (Rienzi and Ubaldi, 2015). Ovarian tissue cryopreservation was proven to be effective in cancer patients but has been described only in a few case reports for patients with endometriosis (Donnez *et al.*, 2005; Oktay and Oktem, 2010). This innovative technique is not particularly suitable as a standard approach for patients with endometriosis, a benign disease in which ovarian surgery is conservative in most cases, and the removal of normal cortical tissue for fertility preservation reasons can further deteriorate ovarian reserve. However, it could be applied in selected situations; for example, the possibility proposed by Donnez *et al.* of cryopreserving healthy fragments of ovarian tissue surgically removed from around the endometrioma or from an ovary removed when the severity of the disease makes oophorectomy mandatory (Donnez *et al.*, 2005).

In conclusion, it is important to emphasize that, based on available data, fertility preservation for patients with endometriosis should be performed using oocyte vitrification rather than embryo vitrification or ovarian tissue cryopreservation.

### Proposed algorithm for fertility preservation in endometriosis patients

**Figure 1.**
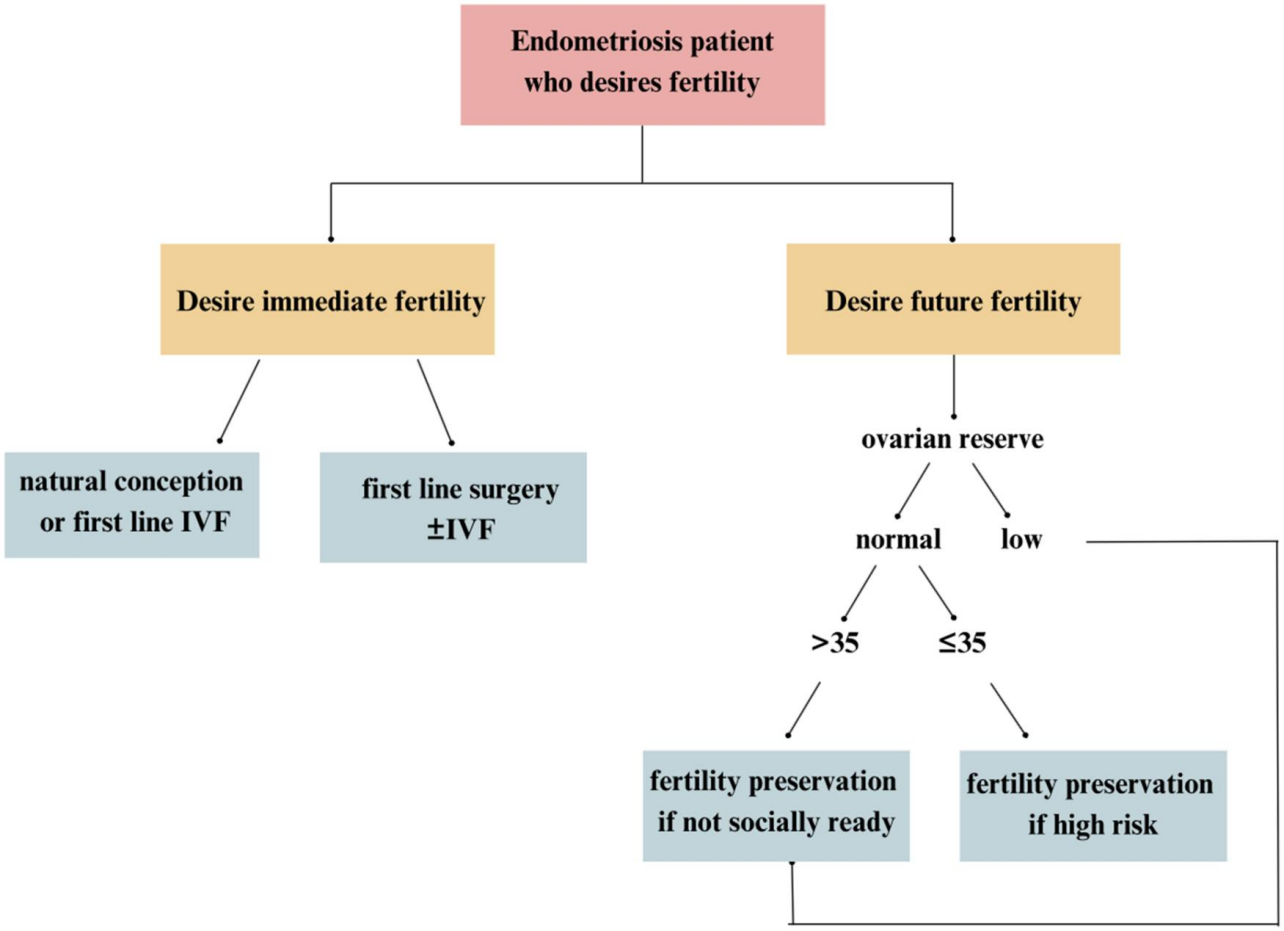
**Proposed algorithm for the management of fertility preservation in women with endometriosis**. The algorithm described focuses on managing a patient who wishes to preserve fertility, and it is divided into two main pathways based on the patient’s preferences. If the patient desires immediate pregnancy, two options are offered: the first involves attempting to conceive naturally or undergoing first-line treatment with IVF. The second option involves an initial surgical approach if there are medical conditions that could benefit from surgery. In this scenario, surgery is considered the first line of treatment, followed by IVF if natural conception is not achieved. If the patient wishes to preserve fertility for the future and does not wish to conceive in the short term, an evaluation of ovarian reserve is required. If the ovarian reserve is low, immediate fertility preservation is recommended, particularly if the patient is not socially ready to conceive. Low ovarian reserve refers to a reduced quantity or quality of oocytes, assessed using markers like AMH, AFC, and age. If the ovarian reserve is normal, the algorithm calls for an assessment of age: if the patient is over 35, fertility preservation is advised if they are not socially ready to conceive. If under 35, fertility preservation is only considered if there are high-risk factors, such as severe endometriosis, prior ovarian surgeries, or conditions that significantly threaten fertility. This personalized approach ensures the treatment pathway is tailored to the patient’s clinical conditions and reproductive goals. AMH: anti-Müllerian hormone; AFC: antral follicle count.

We propose an algorithm for the management of fertility preservation in women with endometriosis (see Fig. 1) tailored to the patient’s specific needs and reproductive goals. The figure provides a structured yet flexible approach to guide decision-making, whether the patient is seeking immediate pregnancy or planning for future fertility. To clarify, the term ‘low ovarian reserve’ refers to a condition where the number or quality of available oocytes is diminished. This is typically evaluated through markers such as AMH levels, AFC, and the patient’s age. A low ovarian reserve is associated with a reduced likelihood of achieving pregnancy, either naturally or through ART. Similarly, high risk in this context refers to situations where factors such as the progression of endometriosis, the natural decline in fertility with age, or other medical conditions such as prior ovarian surgeries pose a significant threat to the patient’s ability to conceive in the future. This includes more severe cases of the disease, such as ovarian endometriomas or extensive adhesions, as well as a poor hormonal response or a history of failed fertility treatments.

## Conclusions

Endometriosis is a chronic disease that can compromise fertility in 30–50% of affected patients. The decision between primary surgery and ART can be challenging, and the therapeutic strategy must be customized according to the clinical characteristics and the needs of the patients (i.e. age, ovarian reserve, type and severity of endometriosis lesions, presence of symptoms, history of surgery, patient desire for pregnancy) (Table 2). For example, younger patients experiencing more severe symptoms may derive greater benefits from surgery, whereas older patients might prefer ART to enhance immediate fertility outcomes (Working group of ESGE, ESHRE, and WES, 2020).

**Table 2.** Key criteria for effective fertility preservation.

Criteria	Description
Patient desire	The patient’s desire is a key factor in deciding personalized strategies.
Age	The patient’s age plays a crucial role in fertility preservation.
Ovarian reserve	Ovarian reserve is critical for assessing fertility options.
First-line surgery	First-line surgery can influence fertility outcomes.

Personalized approaches, considering age and ovarian reserve, are crucial for effective fertility preservation, especially when surgery is involved.

However, considering the detrimental effect of endometriosis on ovarian reserve due to surgical intervention, which may be necessary, or the pathology *per se*, endometriosis has been highlighted as a condition that may require fertility preservation procedures.

Reported experiences regarding the feasibility and success of fertility preservation in patients with endometriosis have significantly increased in recent medical literature. In particular, oocyte cryopreservation seems to be the most suitable technique for young patients with endometriosis. Age and residual ovarian reserve are confirmed to be key factors in retrieving sufficient oocytes, while previous ovarian surgery seems to negatively affect the outcomes in terms of recovered oocytes.

However, also given the difficulty in predicting which patient will develop endometriosis-related infertility and the severity of ovarian damage following the development of endometriosis lesions or surgical treatment, homogeneous data regarding indications for fertility preservation are lacking in the scientific literature. The main proposed indications for the preservation regard women with bilateral endometriomas, those operated unilaterally with a contralateral recurrence, and those with preoperative reduced ovarian reserve. These patients certainly should be advised about the risk of ovarian reserve damage and can be informed about the possibility of cryopreserving oocytes with a personalized counseling. Future studies are needed to develop a consensus about the clinical characteristics of patients to include in fertility preservation programmes and to better understand the cost-effectiveness of the procedure, to improve the accuracy of the counseling and the correct management of such a complex disease.

## Data Availability

This study is a narrative review, and no new data were generated in this work.
